# Treatment of acquired drug resistance in multiple myeloma by combination therapy with XPO1 and topoisomerase II inhibitors

**DOI:** 10.1186/s13045-016-0304-z

**Published:** 2016-08-24

**Authors:** Joel G. Turner, Jana L. Dawson, Steven Grant, Kenneth H. Shain, William S. Dalton, Yun Dai, Mark Meads, Rachid Baz, Michael Kauffman, Sharon Shacham, Daniel M. Sullivan

**Affiliations:** 1 Chemical Biology and Molecular Medicine Program, H. Lee Moffitt Cancer Center & Research Institute, Tampa, FL USA; 2 Massey Cancer Center, Virginia Commonwealth University, Richmond, VA USA; 3 Department of Malignant Hematology, H. Lee Moffitt Cancer Center & Research Institute, Tampa, FL USA; 4 M2Gen® Biotechnologies, H. Lee Moffitt Cancer Center & Research Institute, Tampa, FL USA; 5 Karyopharm Therapeutics, Natick, MA USA; 6 Department of Blood and Marrow Transplantation, H. Lee Moffitt Cancer Center & Research Institute, Tampa, FL USA; 7 H. Lee Moffitt Cancer Center & Research Institute, 12902 Magnolia Drive, Tampa, FL 33612 USA

**Keywords:** Acquired drug resistance, Liposomal doxorubicin, Multiple myeloma, Mouse models, Relapsed/refractory myeloma, XPO1 inhibition

## Abstract

**Background:**

Acquired drug resistance is the greatest obstacle to the successful treatment of multiple myeloma (MM). Despite recent advanced treatment options such as liposomal formulations, proteasome inhibitors, immunomodulatory drugs, myeloma-targeted antibodies, and histone deacetylase inhibitors, MM is still considered an incurable disease.

**Methods:**

We investigated whether the clinical exportin 1 (XPO1) inhibitor selinexor (KPT-330), when combined with pegylated liposomal doxorubicin (PLD) or doxorubicin hydrochloride, could overcome acquired drug resistance in multidrug-resistant human MM xenograft tumors, four different multidrug-resistant MM cell lines, or ex vivo MM biopsies from relapsed/refractory patients. Mechanistic studies were performed to assess co-localization of topoisomerase II alpha (TOP2A), DNA damage, and siRNA knockdown of drug targets.

**Results:**

Selinexor was found to restore sensitivity of multidrug-resistant 8226B25, 8226Dox6, 8226Dox40, and U266PSR human MM cells to doxorubicin to levels found in parental myeloma cell lines. NOD/SCID-γ mice challenged with drug-resistant or parental U266 human MM and treated with selinexor/PLD had significantly decreased tumor growth and increased survival with minimal toxicity. Selinexor/doxorubicin treatment selectively induced apoptosis in CD138/light-chain-positive MM cells without affecting non-myeloma cells in ex vivo-treated bone marrow aspirates from newly diagnosed or relapsed/refractory MM patients. Selinexor inhibited XPO1-TOP2A protein complexes (proximity ligation assay), preventing nuclear export of TOP2A in both parental and multidrug-resistant MM cell lines. Selinexor/doxorubicin treatment significantly increased DNA damage (comet assay/γ-H2AX) in both parental and drug-resistant MM cells. TOP2A knockdown reversed both the anti-tumor effect and significantly reduced DNA damage induced by selinexor/doxorubicin treatment.

**Conclusions:**

The combination of an XPO1 inhibitor and liposomal doxorubicin was highly effective against acquired drug resistance in in vitro MM models, in in vivo xenograft studies, and in ex vivo samples obtained from patients with relapsed/refractory myeloma. This drug combination synergistically induced TOP2A-mediated DNA damage and subsequent apoptosis. In addition, based on our preclinical data, we have initiated a phase I/II study with the XPO1 inhibitor selinexor and PLD (ClinicalTrials.gov NCT02186834). Initial results from both preclinical and clinical trials have shown significant promise for this drug combination for the treatment of MM.

## Background

In cancer cells, the location of a tumor-suppressive or oncogenic protein within the cell is as important as its expression. We have shown that cancer cells utilize the process of nuclear-cytoplasmic transport through the nuclear pore complex to effectively evade anti-cancer mechanisms [1–5]. In our previous studies, we have shown that knockdown of exportin 1 (XPO1) protein by siRNA or inhibition with an XPO1 inhibitor will sensitize drug-resistant myeloma cells to the topoisomerase II (TOP2) inhibitor doxorubicin [3, 5]. We have also shown that XPO1 inhibitors are able to prevent nuclear export and promote nuclear accumulation of the tumor suppressor protein p53, and prevent the export of the drug target TOP2A [3, 5]. In addition, XPO1 inhibitors were found to reverse de novo drug-resistance of multiple myeloma (MM) cells in high-density cell culture models and drug resistance conferred to MM cell lines when co-cultured with bone marrow stromal cells [5].

Recent publications have indicated that XPO1 inhibitors, particularly the orally available clinical compound selinexor (KPT-330), may be effective against various malignancies, including breast cancer [6, 7], glioblastoma [8], hepatocellular carcinoma [9], kidney cancer [10, 11], leukemia [12–16], lung cancer [17], mantle cell lymphoma [18, 19], melanoma [20, 21], mesothelioma [22], non-Hodgkin lymphoma [23], ovarian cancer [7], pancreatic cancer [24, 25], prostate cancer [25, 26], and MM [5, 13, 27].

Recent studies in MM have shown that XPO1 protein levels are increased in plasma cells from newly diagnosed MM patients when compared with normal plasma cells [13, 27] or with plasma cells from those with monoclonal gammopathy of undetermined significance and smoldering MM [27]. In addition, high levels of XPO1 may be associated with decreases in event-free and overall survival in MM [13]. When treated with XPO1 inhibitors, 21 different human MM cell lines were found to have decreased cell viability [3, 5, 13, 27]. XPO1 inhibitors in MM have been shown to dysregulate the following cancer-related proteins or mRNAs: c-myc, CDC25A, BRD4, p53, Mcl-1, BCl-xL, NF-kB, p21, p27, IkB, FOXO3A, FOXO1A, PP2A, PUMA, BAX, CHOP, C1-0orf10, MIC1, IL-6, VEGF, MIP1ß, and IL-10 [5, 13, 27].

What has not been addressed in previous studies is whether XPO1 inhibitors are effective in overcoming acquired drug-resistant MM phenotypes, which develop in patients during treatment with standard of care therapies. In patients with MM, acquired drug resistance is a major obstacle, as the disease is considered incurable despite significant advances afforded by immunomodulatory drugs (thalidomide, lenalidomide, pomalidomide), proteasome inhibitors (bortezomib, carfilzomib, ixazomib), antibodies targeting SLAMF7 protein (elotuzumab) and CD-38 (daratumumab), histone deacetylase inhibitors (panobinostat), and high-dose chemotherapy with autologous stem cell rescue.

The recent resurgence of doxorubicin as a treatment for multiple myeloma has been observed in the clinic due to its reformulation in poly(ethylene glycol)-coated liposomes. This formulation increases circulation time and has a unique toxicity profile, including mild myelosuppression, minimal alopecia, and no cardiac toxicity. In addition, liposomal doxorubicin accumulates preferentially in tissues with increased microvascular permeability seen in cancers [28–30]. In the present study, we show that XPO1 inhibition sensitized drug-resistant MM cells to liposomal doxorubicin in in vitro and in vivo models and ex vivo in relapsed/refractory patient MM cells, thus demonstrating that this combination may provide the means for overcoming acquired drug resistance in MM.

## Methods

### Cell lines

Human MM cell lines RPMI 8226, U266, and NCI-H929 were obtained from the American Type Culture Collection (ATCC; Manassas, VA). MM cell lines 8226B25 and U266PSR were developed incrementally [31, 32]. The U266PSR cell line expresses a modest increase in Mcl-1 [33] and markedly lower expression of the apoptosis-promoting factor Bim [31, 32], resulting in enhanced cell survival by inhibiting apoptosis. Doxorubicin-resistant 8226Dox6 and 8226Dox40 cell lines were produced by the incremental addition of doxorubicin [34]; these cell lines were found to overexpress the *MDR1* gene, which prevented intracellular accumulation of doxorubicin, resulting in resistance [35, 36]. All cell lines were authenticated by the Moffitt Cancer Center Molecular Genomics Core Facility according to ATCC guidelines [37].

### Drug-resistant cell lines treated with XPO1 inhibitors and doxorubicin

Parental 8226 and U226 and drug-resistant 8226B25, 8226Dox6, 8226Dox40, and U266PSR human MM cells were grown at low-density (log growth phase) conditions (3–4 × 10^5^ cells/mL) and cultured for 20 h with either 300 nM selinexor (Karyopharm Therapeutics) or 10 nM KOS-2464 (Bristol-Myers Squibb) with and without 2 μM doxorubicin (Sigma Chemical). Optimal drug concentrations were determined by titration experiments. Cells were fixed in Cytofix/Cytoperm buffer (Becton-Dickinson) and permeabilized in Perm/Wash buffer (Becton-Dickinson), and apoptosis was measured by flow cytometry using anti-activated caspase 3/Alexa Fluor 488 (Cell Signaling Technology) staining.

### Bone marrow aspirate processing and apoptosis assay of patient myeloma cells

Bone marrow aspirates were collected from newly diagnosed (*n* = 19) and relapsed (*n* = 12)/refractory (*n* = 10) patients. Isolated bone marrow mononuclear cells were incubated at 4–8 × 10^6^/mL in 200 μL RPMI (Fisher) containing 10 % FBS in 96-well plates, treated with either selinexor (300 nM) or KOS-2464 (300 nM) with and without 2 μM doxorubicin, and incubated for 20 h in a 5 % CO_2_ humidified incubator. The cells were then fixed and assayed for apoptosis according to methods outlined in Turner et al. [5].

### NOD/SCID-γ mouse studies with selinexor ± PLD

Drug-resistant U266PSR human myeloma cells (10^6^) or parental U266 cells (5 × 10^6^) were injected subcutaneously into flanks of female NOD/SCID-γ mice, and tumors were allowed to grow for 14 days before the start of treatment [31]. Mice were treated with PLD (0.5 mg/kg) once weekly by intraperitoneal injection, by oral gavage with selinexor (10 mg/kg) twice weekly, or with the combination where selinexor treatment was followed 1–2 h later by PLD injection. Five mice were used per experimental group. Tumors were measured by calipers, and tumor volumes (mm^3^) were calculated by the formula (length × width^2^)/2. Animals were killed upon achieving a tumor volume >2000 mm^3^ or if the mouse lost >15 % of its body weight; this was used to define survival. Drug toxicity was assayed by mouse weights with a decrease of ≥10 % considered an indication of toxicity by the drug regimen.

### Proximity ligation assay

Log-phase H929, 8226, 8226B25, 8226Dox6, U226, and U226PSR MM cells were placed at plateau conditions (4 × 10^6^ cells/mL) and treated with 300 nM selinexor for 4 h. Cells were washed with PBS, and cytospins were made at 1 × 10^5^ cells/slide and fixed with 4 % paraformaldehyde/PBS and permeabilized with 0.5 % Triton X-100. Slides were blocked in 2 % BSA/PBS and incubated with primary antibodies to TOP2A (Kis1; Millipore) and XPO1 (H-300; Santa Cruz). Proximity ligation assay was performed according to the manufacturer’s protocol (Olink Bioscience, Uppsala, Sweden) [38]. A red fluorescent signal was generated only when XPO1 and TOP2A were in close proximity (<40 nm). 4′,6-Diamidino-2-phenylindole (DAPI) was used to stain the nuclei. Samples were observed with a Leica TCS SP5 AOBS laser scanning confocal microscope. The total number of foci per nucleus and cell were analyzed for number and area. This experiment was repeated three times. Western blots were made of the selinexor-treated cells at 4 h for XPO1 and TOP2A protein expression levels.

### Neutral comet assay

Drug-resistant 8226B25, 8226Dox6, and U266PSR and parental 8226 and U226 cells grown under log-phase conditions were placed at high-density conditions (2–4 × 10^6^ cells/mL), and selinexor (100 nM) was added to media for 20 h followed by the addition of 5 μM doxorubicin for 1 h. Neutral comet assay to determine DNA damage was performed according to the manufacturer’s protocol (Trevigen, Gaithersburg, MD) [39, 40]. Proteinase K was added to digest any protein-associated DNA damage caused by topoisomerase-mediated formation of cleavable complexes, indicating that DNA damage was due to double-strand breaks produced from TOP2 [41]. Electrophoresis was performed at room temperature in TBE buffer at a constant 0.85 V/cm for 15 min. Slides were rinsed very briefly with ddH_2_O and then fixed in 70 % ethanol for 5 min. Slides were air-dried and then stored at room temperature in a slide box containing desiccant until comets were stained and viewed by microscopy. Comets were stained with SYBR green (Molecular Probes) diluted at 1:10,000 in TBE. Images were taken with ×40 objective lens using a fluorescein filter. The Loats Associates Incorporated Comet Analysis System was used to calculate tail moment [42], which is the product of distance and normalized intensity integrated over the tail length. This is a damage measurement combining the amount of DNA in the tail with the distance of migration (severity of damage). A minimum of 50 comets were analyzed for each dose, and each experiment was repeated three times.

### Gamma-H2AX and TOP2A knockdown

Cells were treated with single-agent doxorubicin (2 μM), selinexor (300 nM), and their combination for 20 h and then assayed for phospho-H2AX (Ser139) expression (JBW301, FITC conjugate, Millipore) by flow cytometry (*n* = 4). To determine if apoptosis and DNA strand breaks are dependent on the presence of TOP2A, H929 MM cells were transfected by electroporation with a TOP2A-specific siRNA (Silencer Select Validated cat. #4390824, Ambion). Electroporation of MM cells was accomplished by a method outlined in Turner et al. [3]. A control group of cells was transfected with a non-coding siRNA. Transfected (TOP2A knockdown and control siRNA) H929 MM cells were then incubated with selinexor (300 nM) +/− doxorubicin (2 μM) for 20 h and assayed by flow cytometry for DNA double-strand breaks using phospho-H2AX (Ser139) expression (JBW301) and apoptosis by activated-caspase 3 (ASP175, AF488, Cell Signaling).

## Results

### XPO1 inhibition sensitizes drug-resistant MM cell lines to doxorubicin

**Fig. 1:**
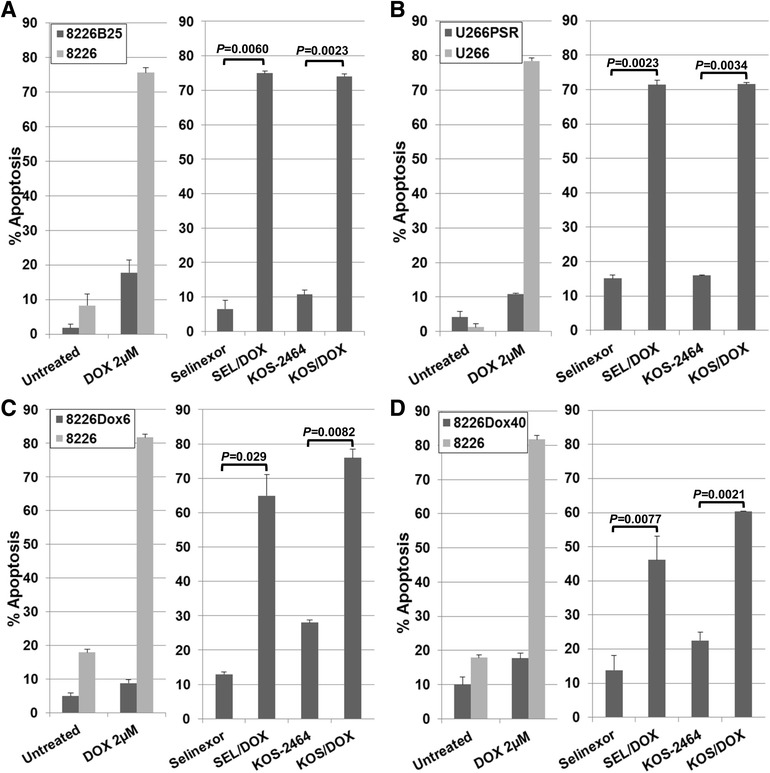
XPO1 inhibition sensitizes drug-resistant human MM cell lines to doxorubicin (DOX). Human 8226B25 (**a**), U266PSR (**b**), 8226Dox6 (**c**), and 8226Dox40 (**d**) drug-resistant and parental MM cell lines were treated concurrently for 20 h with selinexor (300 nM) or KOS-2464 (10 nM) +/− doxorubicin (2 μM) and assayed for apoptosis by flow cytometry (activated caspase 3) (*n* = 3). XPO1 inhibitors sensitized drug-resistant cells to DOX compared with single-agent treatment

Apoptosis results (flow cytometry using activated caspase 3) in human drug-resistant and parental MM cells after 20-h concurrent treatment with selinexor (300 nM) or KOS-2464 (10 nM) ± doxorubicin (2 μM) are shown in Fig. 1. Both U266 and 8226 parental cell lines were highly sensitive to single-drug treatment with doxorubicin. Drug-resistant 8226B25 (Fig. 1a) and U266PSR MM cell lines (Fig. 1b) and doxorubicin-resistant 8226Dox6 (Fig. 1c) and 8266Dox40 MM cell lines (Fig. 1d) were resistant to single-agent doxorubicin (5.9-, 11.6-, 16.7-, and 10.7-fold, respectively) compared with parental cells. The XPO1 inhibitor selinexor sensitized drug-resistant human MM 8226B25 (*P* = 0.0060), U266PSR (*P* = 0.0023), 8226Dox6 (*P* = 0.029), and 8226Dox40 (*P* = 0.0077) cell lines to doxorubicin compared with single-agent treatment with doxorubicin alone. In addition, KOS-2464 also sensitized drug-resistant human MM 8226B25 (*P* = 0.0023), U266PSR (*P* = 0.0034), 8226Dox6 (*P* = 0.0.029), and 8226Dox40 (*P* = 0.0077) cell lines to doxorubicin compared with single-agent treatment with doxorubicin alone (Fig. 1a–d).

### Ex vivo treatment of newly diagnosed, relapsed/refractory patient MM cells with KOS-2464 and selinexor sensitizes cells to doxorubicin

CD138^+^/light chain^+^ MM cells were isolated from bone marrow aspirates from newly diagnosed (*n* = 19) and relapsed (*n* = 12) or refractory (*n* = 10) patients. The relapsed/refractory patients had received therapies that included an average of 4.7 regimens of chemotherapy, including two or more of the following drugs or drug combinations: busulfan, carfilzomib, cytoxan, dexamethasone, elutozumab, pegylated liposomal doxorubicin (PLD or Doxil), panobinostat, pomalidomide, prednisone, revlimid (lenalidomide), velcade (bortezomib), and melphalan with autologous stem cell transplantation.

**Fig. 2:**
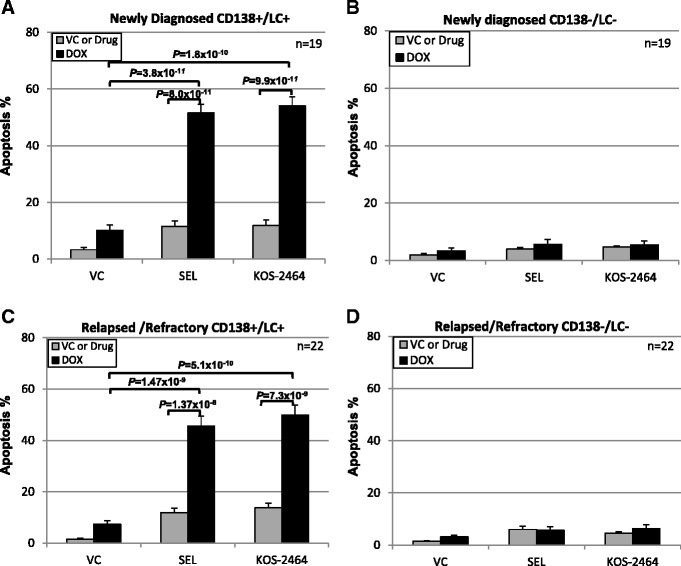
Selinexor and KOS-2464 sensitize newly diagnosed and relapsed patient MM cells to doxorubicin. Bone marrow mononuclear cells were isolated and treated with selinexor or KOS-2464 +/− doxorubicin and fluorescently labeled with antibodies against activated caspase 3, CD138, and light chain (kappa or lambda). Newly diagnosed (*n* = 19) and relapsed (*n* = 22) CD-138/light-chain double-positive MM patient samples were all sensitized by selinexor and KOS-2464 to doxorubicin (*P* ≤ 1.37 × 10^−8^) versus single-agent treatment as shown by increased apoptosis (**a**, **c**). Non-myeloma CD138/light-chain double-negative patient cells were not sensitized to apoptosis by XPO1 inhibitors (**b, d**)

Both newly diagnosed and relapsed/refractory MM patient cells were sensitized by selinexor (*P* = 8.0 × 10^−11^ and *P* = 1.37 × 10^−8^, respectively) and KOS-2464 (*P* = 9.9 × 10^−11^ and *P* = 7.3 × 10^−9^, respectively) to doxorubicin (Fig. 2a, c) as shown by activated caspase 3 staining and detection by flow cytometry. In contrast, CD138/light-chain double-negative non-myeloma cells from the same patient bone marrow aspirates were not sensitized to apoptosis by XPO1 inhibitors (Fig. 2b, d).

### In vivo NOD/SCID-γ mouse studies with selinexor and PLD

**Fig. 3:**
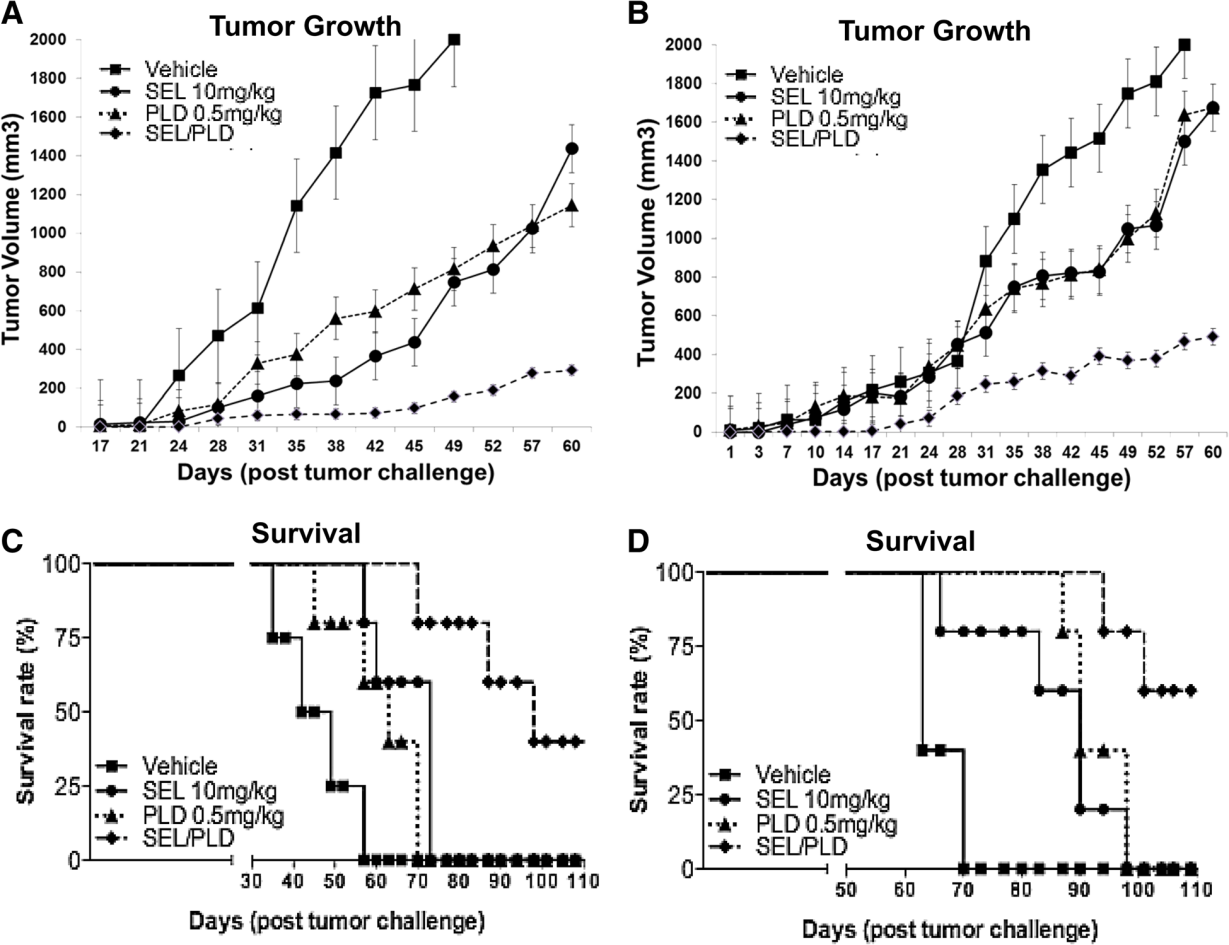
In vivo studies. PLD/selinexor treatment reduced drug-resistant U266PSR (**a**) and parental U266 (**b**) tumor growth compared with single-agent treatment with PLD (*P* = 0.001) or selinexor (*P* = 0.009) in drug-resistant U266PSR (**a**) and PLD (*P* = 0.0003) or selinexor (*P* = 0.0079) in parental U266 (**b**). **c, d**: Selinexor/PLD significantly improved survival in U266PSR-challenged mice compared with single-agent selinexor (*P* = 0.0095) or PLD (*P* = 0.0018) (**c**) and in U266 compared with selinexor (*P* = 0.0002) or PLD (*P* = 0.0024) (**d**)

In mouse studies, drug-resistant U266PSR human MM cells and parental U266 cells controls were used. U266PSR cells have been shown to be approximately seven- to eightfold more resistant to doxorubicin than U226 parental cells (Fig. 1b) [31–33]. As shown in Fig. 3b, PLD combined with selinexor reduced parental U266 tumor growth versus single-agent PLD (*P* = 0.0003) or selinexor (*P* = 0.0079). PLD combined with selinexor also reduced drug-resistant U266PSR tumor growth (Fig. 3a) versus single-agent PLD (*P* = 0.001) or selinexor (*P* = 0.009). Selinexor/PLD significantly improved survival in U266-challenged mice when compared with single-agent selinexor (*P* = 0.0002) or PLD (*P* = 0.0024) (Fig. 3d) and in U266PSR when compared with selinexor (*P* = 0.0095) or PLD (*P* = 0.0018) (Fig. 3c). At the end of the study (4 months), 40 % of the U266PSR-challenged mice and 60 % of the U266 parental MM-challenged mice had survived; all untreated control, single-agent PLD-treated, and selinexor-treated mice were euthanatized due to tumor size (>2000 mm^3^) before the end of the study. Toxicity, assessed by weight loss greater than 10 %, was less than 2 % in all treatment groups.

### Selinexor inhibits XPO1-TOP2A binding

**Fig. 4:**
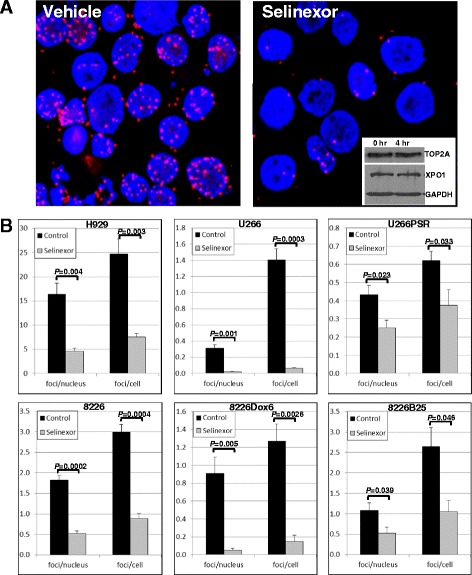
Selinexor inhibits XPO1-TOP2A binding. Parental H929, U266, and 8226 and drug-resistant U266PSR, 8226Dox6, and 8226B25 plateau-density human MM cells (3 × 10^6^/ml) were treated with selinexor (300 nM), cytospun, and assayed for intracellular co-localization of XPO1 and TOP2A by proximity ligation assay. A red fluorescent signal was generated only when XPO1 and TOP2A were in close proximity (<40 nm). **a** Selinexor blocked proximity co-localization of XPO1 and TOP2A. *Inset*, selinexor treatment did not affect XPO1 or TOP2A protein levels at 4 h as shown by Western blot. **b** Analysis of the number of XPO1-TOP2A foci showed that selinexor significantly decreased the number of foci in the nucleus and whole cells of both parental and drug-resistant MM cells

To determine whether selinexor specifically inhibits XPO1-TOP2A binding, proximity ligation assays were performed on parental 8226, H929, and U266 MM cell lines and drug-resistant 8226B25, 8226Dox6, and U226PSR cell lines (Fig. 4). Selinexor blocked proximity co-localization of XPO1 and TOP2A as shown by the number of XPO1-TOP2A foci (Fig. 4a). Selinexor significantly decreased the number of foci in the nucleus and whole cells (Fig. 4b) in H929 (*P* ≤ 0.004), U266 (*P* ≤ 0.001), U266PSR (*P* ≤ 0.033), 8226 (*P* ≤ 0.0004), 8226Dox6 (*P* ≤ 0.005), and 8226B25 (*P* ≤ 0.046) cells. XPO1 and TOP2A protein levels were unaffected by selinexor treatment for up to 4–6 h (Fig. 4a, inset). Selinexor covalent modification of the XPO1 binding site precluded XPO1 binding the nuclear export signal of TOP2A, thus preventing the nuclear export of TOP2A and increasing the amount of nuclear enzyme.

### XPO1 inhibitors increased DNA damage when used with doxorubicin

**Fig. 5:**
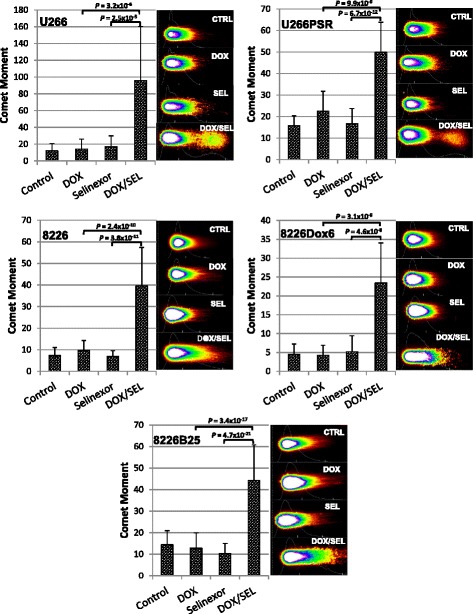
Comet DNA fragmentation assay. High-density human 8226 and U226 and drug-resistant cell lines 8226Dox6, 8226B25, and U266PSR MM (2 × 10^6^/mL) were treated with selinexor (100 nM) followed by doxorubicin (5 μM). DNA fragmentation (double-strand breaks) was measured by the neutral comet assay. Doxorubicin or selinexor, when used as single agents, increased DNA double-strand cleavage compared with untreated controls. However, selinexor + doxorubicin further increased DNA fragmentation over doxorubicin or selinexor alone

Human 8226 MM cells (2 × 10^6^/mL) were treated with selinexor (100 nM) for 20 h followed by doxorubicin (5 μM) for an additional 1 h, and DNA fragmentation was measured by the neutral comet assay (Fig. 5). Selinexor/doxorubicin treatment increased DNA damage over single-agent doxorubicin or selinexor in U266 (*P* = 3.2 × 10^−6^/*P* = 2.5 × 10^−5^), PSR (*P* = 9.9 ×1 0^−9^/*P* = 6.7 × 10^−12^), 8226 (*P* = 2.4 × 10^−10^/*P* = 3.8 × 10^−11^), 8226Dox6 (*P* = 3.1 × 10^−9^/*P* = 4.6 × 10^−8^), and B25 (*P* = 3.4 × 10^−17^/*P* = 4.7 × 10^−21^) MM cell lines (*n* = 50 comets each per cell line and treatment condition for three experiments each) (Fig. 5).

### DNA damage and apoptosis induced by selinexor/doxorubicin is TOP2A dependent

**Fig. 6:**
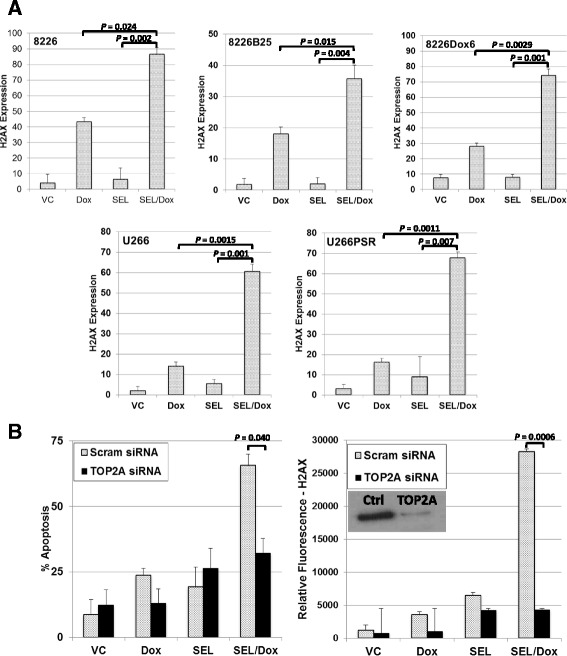
Mechanism for selinexor and doxorubicin synergy. **a** Parental MM cell lines 8226 and U226 and drug-resistant 8226B25, 8226Dox6, and U266PSR cells were treated with doxorubicin (2 μM), selinexor (300 nM), or the combination for 20 h. Both parental and drug-resistant cell lines, when treated with the combination of selinexor and doxorubicin, had significantly increased DNA damage as shown by increased phospho-H2AX (Ser139) expression (*n* = 4). **b** SiRNA knockdown of TOP2A resulted in a substantial decrease in DNA damage (phospho-H2AX (Ser139)) and apoptosis, suggesting that selinexor and doxorubicin induced DNA damage, and subsequent apoptosis is TOP2A dependent. *Inset*, TOP2A knockdown (Western blot)

DNA damage was measured by phospho-H2AX expression in selinexor- and doxorubicin-treated cells. 8226, 8226B25, 8226Dox6, U266, and U266PSR human MM cells were treated concurrently for 20 h and stained for phospho-H2AX expression by flow cytometry (Fig. 6). DNA damage was synergistically induced by the combination of selinexor and doxorubicin compared with single-agent doxorubicin or selinexor in parental 8226 (*P* ≤ 0.024) and U266 (*P* ≤ 0.013) MM cell lines and in drug-resistant 8226B25 (*P* ≤ 0.015), 8226Dox6 (*P* ≤ 0.0027), and U266PSR (*P* ≤ 0.027) MM cell lines (Fig. 6a). Phospho-H2AX levels increased four- to sixfold in selinexor- and doxorubicin-treated cells compared with selinexor or doxorubicin alone. When cells were transfected with a TOP2A-specific siRNA, phospho-H2AX levels decreased significantly (*P* = 0.0006) (Fig. 6b). Apoptosis was also significantly reduced in selinexor- and doxorubicin-treated cells with TOP2A knockdown (Fig. 6b). These data indicate that DNA damage and subsequent cell death induced by selinexor/doxorubicin synergy is TOP2A dependent.

## Discussion

Drug resistance is the greatest obstacle for treatment of MM and many other cancers. In this study, we demonstrated the possibility that acquired drug resistance in MM may be overcome by a combinatorial therapy including the XPO1 inhibitor selinexor with the TOP2 inhibitor doxorubicin. The drug-resistant cell lines used in this study included human MM made resistant to doxorubicin (8226Dox6 and 8226Dox40) and cells made resistant to the proteasome inhibitor bortezomib (8266B25 and U266PSR); however, all of these cell lines have a multidrug-resistant phenotype. The mechanism of drug resistance of 8224Dox6 and 8226Dox40 MM cell lines has been shown to be due to an upregulation of the MDR-1 gene [34–36]. These cells have been shown to be resistant to many chemotherapeutic agents, including doxorubicin, daunorubicin, mitoxantrone, acronycine, etoposide, melphalan, and vincristine. Drug-resistant 8226B25 and U266PSR MM cell lines were developed by the incremental addition of bortezomib to 8226 and U266 parental cell lines. These cells were found to be highly resistant to bortezomib, carfilzomib, doxorubicin, and melphalan and therefore also have a multidrug-resistant phenotype (data not shown). The U266PSR cell line expresses a modest increase in Mcl-1 [33] and markedly lower expression of the apoptosis-promoting factor Bim [31, 32], resulting in enhanced cell survival by inhibiting apoptosis and resulting in multidrug resistance.

The TOP2 poison, doxorubicin, functions by arresting TOP2A in cleavable complexes, resulting in double-strand DNA breaks and subsequent apoptotic cell death of cancer cells [43–45]. Doxorubicin has seen resurgence in the clinic because of the development of new liposomal formulations [28]. Pegylated liposomal doxorubicin provides reduced toxicity, increases in vivo circulation time, and accumulates preferentially in tumors, resulting in increased efficacy in the treatment of MM [28–30]. We demonstrated by proximity ligation assay that TOP2A and XPO1 co-localization is disrupted when MM cells are incubated with selinexor. These data correlate well with previous studies where de novo MM drug resistance to doxorubicin is caused by nuclear export of TOP2A, an effect that is reversed by blocking nuclear export of TOP2A with an XPO1 inhibitor [3, 5, 40, 46]. XPO1 inhibitors such as selinexor prevent export of TOP2A, keeping it in the nucleus and in close proximity to the DNA. We found that, when XPO1 inhibitors are used with doxorubicin or PLD, DNA damage is increased (comet assay/phospho-H2AX), resulting in apoptosis and improving the effectiveness of doxorubicin. In this study, we demonstrated that this two-drug combination is also highly effective against drug-resistant myeloma, in both cell lines and cells derived from patients. siRNA knockdown of TOP2A significantly decreased both DNA damage and apoptosis, confirming this mechanism of action. The synergistic effect seen with selinexor combined with doxorubicin also translated to drug-resistant MM cell lines (in vitro and in vivo) and in MM cells isolated from relapsed/refractory patient bone marrow. Relapsed/refractory patients received multiple rounds of chemotherapy that included an average of 4.7 lines of chemotherapy. These patient cells, like the drug-resistant cell lines, are also highly drug-resistant; however, the combination of selinexor and doxorubicin was effective in the ex vivo treatment of these cells.

Based on these pre-clinical data, we have initiated a phase I/II clinical trial with selinexor and PLD in relapsed/refractory multiple myeloma patients. Preliminary results of the study have been presented at the annual meeting of the American Society of Clinical Oncology; and while the dose escalation continues, encouraging evidence of preliminary efficacy is noted among the 11 response evaluable patients who are heavily pretreated (median of 6 prior lines of therapy) [47]. Specifically two patients had a very good partial response, two patients had a partial response, and another two patients had a minimal response.

## Conclusions

Selinexor, an orally active selective inhibitor of XPO1-mediated nuclear export, is currently undergoing phase 1 and phase 2 studies in a variety of indications, including combinations with PLD and carfilzomib, in both relapsed and refractory MM patients. The results presented in our study support combinatorial clinical trials in relapsed and refractory MM, and perhaps other cancers, that utilize TOP2A therapies.

## Abbreviations

DAPI: 4′,6-Diamidino-2-phenylindole
MM: Multiple myeloma
PLD: Pegylated liposomal doxorubicin
TOP2A: Topoisomerase II alpha
XPO1: Exportin 1
